# Supplementary material for: Boosting AlphaFold protein tertiary structure prediction through MSA engineering and extensive model sampling and ranking in CASP16
Source: Commun Biol. 2025 Nov 17;8:1587. doi: 10.1038/s42003-025-08960-6 (PMC12623963; doi:10.1038/s42003-025-08960-6)
Supplement: Supplementary file 1 — Supplementary Information [file 42003_2025_8960_MOESM1_ESM.pdf]

Supplementary Material

Jian Liu, Pawan Neupane, Jianlin Cheng

September 12, 2025

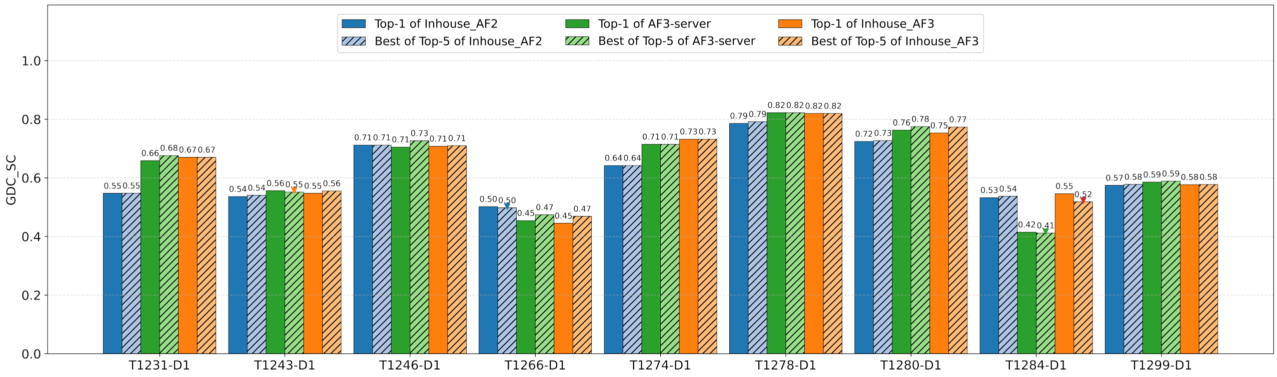

Figure S1: Comparison of GDC_SC (Global Distance Calculation for side chains) performance for 9 single-chain monomer targets with GDT-TS > 0.7, predicted by our in-house AlphaFold2, our in-house AlphaFold3, and the CASP16 AF3-server. For each method, the GDC_SC results are shown for the top-1 model, and for the best-of-top-5 models selected by GDT-TS.

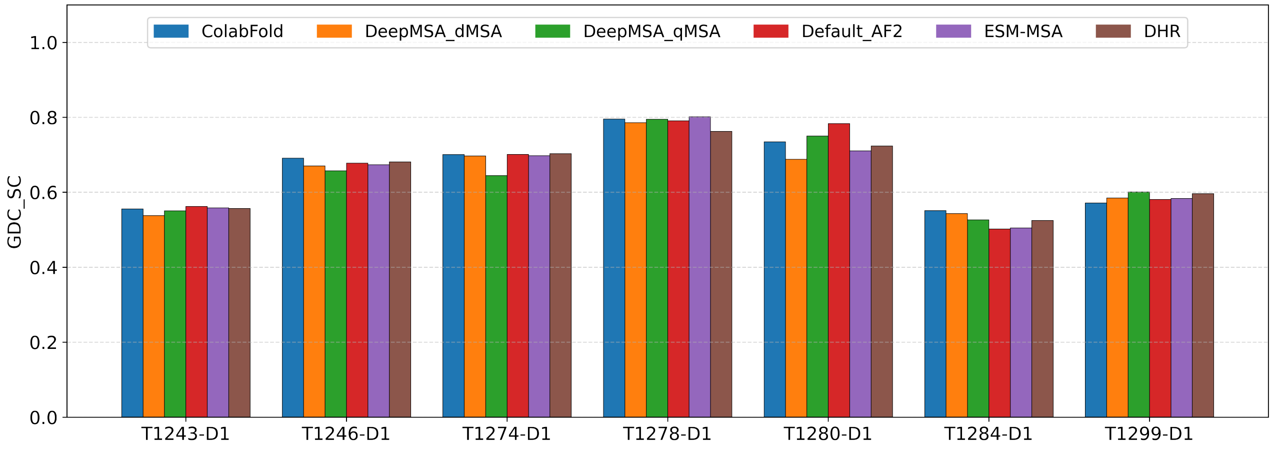

Figure S2: Comparison of GDC_SC performance for top-1 models of 7 single-chain monomer targets (GDT-TS > 0.7) generated by AlphaFold2 using different MSA sources. All models were produced under identical input settings.
