# Supplementary material for: Boosting AlphaFold protein tertiary structure prediction through MSA engineering and extensive model sampling and ranking in CASP16
Source: Commun Biol. 2025 Nov 17;8:1587. doi: 10.1038/s42003-025-08960-6 (PMC12623963; doi:10.1038/s42003-025-08960-6)
Supplement: Supplementary file 2 — Description of Additional Supplementary Materials [file 42003_2025_8960_MOESM2_ESM.pdf]

Description of Additional Supplementary Files

File name: Supplementary Data 1

Description: Source data for the figures
